# Hydrothermal Synthesis of Chitosan and Tea Tree Oil on Plain and Satin Weave Cotton Fabrics

**DOI:** 10.3390/ma15145034

**Published:** 2022-07-20

**Authors:** Sandra Flinčec Grgac, Tea Tesla, Ivana Čorak, Franka Žuvela Bošnjak

**Affiliations:** Faculty of Textile Technology, University of Zagreb, Prilaz baruna Filipovi´ca 28a, 10000 Zagreb, Croatia; tea.tesla97@gmail.com (T.T.); ivana.corak@ttf.unizg.hr (I.Č.); franka.zuvela.bosnjak@ttf.unizg.hr (F.Ž.B.)

**Keywords:** cotton, chitosan, tea tree oil, in situ hydrothermal synthesis, hydrophilicity, antimicrobial effect

## Abstract

The paper aimed at enhancing the antimicrobial activity of chitosan by using tea tree essential oil with the purpose of durably finishing cotton fabrics for use in a hospital environment. The influence of crosslinkers and catalysts on the possibility of obtaining stable bonds using hydrothermal in situ synthesis between cellulosic material and chitosan with and without tea tree essential oil was investigated in detail. The morphology of the sample surface before and after the treatment and textile care cycle was investigated using a field emission scanning electron microscopy (FE-SEM) and indicated the presence of chitosan and a thin film on all treated samples, which showed durability of the treatment. The FTIR spectra obtained by Fourier transform infrared spectroscopy (FTIR) using attenuated total reflection measurement technique (ATR) analysis, showed that all the samples tested recorded physicochemical changes in the structure. The analysis of the samples on the goniometer proved the hydrophilicity of the materials, with a film forming on the surface of the treated samples, which is extremely beneficial given the end use of dressing samples to promote wound healing. The presence of a significant amount of bound chitosan with tea tree oil was confirmed by measuring the mass per unit area of the samples after the treatment and textile care cycles. The results of antimicrobial efficacy show that the materials treated with chitosan were resistant to bacteria and fungi in most cases, but only the samples treated in Bath I showed a zone of inhibition against the fungus *Candida albicans*, indicating the positive effect of tea tree essential oil.

## 1. Introduction

We live in a time where the requirements for maintaining a healthy, safe, and pleasant environment, as well as for protecting against infections caused by pathogenic microorganisms are becoming increasingly stringent [1,2,3]. Therefore, there is always a high demand for medical textiles with antimicrobial properties. Numerous chemicals are used to achieve antimicrobial properties on textiles, including organometals, inorganic salts, phenols and thiophenols, heterocyclic compounds with anionic groups, antibiotics, urea, and similar urea-based compounds, nitro compounds, amines, etc. However, many of these substances are toxic to human health and cannot be easily degraded in nature [1,2]. In the last decade, research on the possibility of using chitosan in textiles intensified.

Chitin is the second most important biopolymer after cellulose, with the production of over 100 million tons per year. Chitosan, a linear biopolymer of glucosamine monomers and small amounts of N-acetyl-glucosamine monomers, is obtained by alkaline N-deacetylation of chitin and is considered to be of natural origin. Due to its excellent biocompatibility, biodegradability, and non-toxicity, it is used in the pharmaceutical industry, e.g., for drug delivery and wound healing, in agriculture, genetic engineering, food industry, environmental control, water purification, paper production, photography, textile industry, etc. [1,4,5,6,7,8,9]. In textile finishing processes, chitosan is most commonly applied to cotton, polyester, and wool fibres, and exhibits an antimicrobial effect on a variety of microorganisms, including fungi, algae, and some bacteria [10,11]. When it comes to Gram-negative and Gram-positive bacteria, there are contradictory opinions in the studies. While some suggest that Gram-positive bacteria are more sensitive to its action due to the presence of polyanionic teichoic acids on the surface, others cite the role of hydrophobic interactions of exposed lipopolysaccharides and claim that Gram-negative bacteria are more significantly affected by chitosan action [12,13]. It is, however, important to emphasise that chitosan has GRAS status (generally recognised as safe).

The antimicrobial activity of chitosan depends on the type of chitosan used, the degree of deacetylation, and the molecular weight [4]. The antimicrobial property of the treated textile is due to the charged amino group of chitosan interacting with the cell wall, leading to the breakdown of proteins and intracellular constituents, thus causing cell death. The higher the degree of deacetylation of chitosan, the more -NH_3_^+^ is transferred, and the stronger the force acting on the electronegative materials on the surface of the bacteria, which improves the bacteriostatic effect. Treated textiles show a clear zone of inhibition against bacterial strains of *E. coli* and *S. aureus* [1,4,14,15,16,17]. The main disadvantage of chitosan as an antimicrobial agent for textiles is its difficult handling and the strong dependence of its activity on the pH and temperature. Since chitosan is only soluble in an acidic medium and becomes a polycation when the pH is lower than pKa (6.3–6.5), a stronger antibacterial effect is observed under acidic conditions [10]. Additionally, homogeneity and degree of deacetylation are some of the factors that increase the solubility of chitosan if the pH is lower than six. Furthermore, during storage and depending on the temperature, some specific properties of chitosan may change, such as viscosity and molecular weight, resulting in varying biocidal efficacy [18].

In the course of previous research, Flinčec Grgac and colleagues investigated various possibilities of implementing chitosan in the structure of cellulosic materials and their mixtures with polyester. It was concluded that the binding of chitosan to and in the cellulose structure, as well as the polyester component in the mixtures, was more stable using the opening of the fibre structure by the mercerization process in NaOH and impregnation with chitosan gel prepared with citric acid, compared to the application of solid chitosan particles in the mercerization process [19,20,21,22].

Considering all the findings related to the use of chitosan in finishing cellulose fabrics, the influence of crosslinkers and catalysts on the possibility of obtaining stable bonds by hydrothermal in situ synthesis between cellulosic material and chitosan with and without tea tree essential oil was investigated. Future research will investigate the use of tea tree essential oil in different concentrations, with the aim of achieving better antimicrobial properties for textile materials. The materials thus developed will be used in further research with the purpose of monitoring their effect on wound healing and skin treatment in humans and animals.

## 2. Materials and Methods

### 2.1. Materials

For this study, 100% cotton fabrics in plain and satin weaves were used. Thread density of the plain weave fabric was 20 picks/cm and 35.8 ends/cm. Mass per unit area of the samples in the plain weave was 158.6 g/m^2^.

Thread density of the satin weave fabric was 26 picks/cm and 35.8 ends/cm. Mass per unit area of the initial samples was 178.5 g/m^2^.

The chitosan, provided by Tricomed SA, Łód’z, Poland, used in the research had the average molecular weight (Mn) of 80,000 Da with a degree of deacetylation (DDA) of 90%. The diameter of the chitosan particles ranged from 1 to 0.5 µm.

Homogenization of the dissolved components (Table 1) was carried out for 15 min at 800 rpm using a magnetic stirrer. The pH of Bath I was five and Bath II was four. In situ synthesis was performed in an autoclave at a temperature of 80 °C for 24 h. The treated fabric specimen was dried at 100 °C for 2 min and finally cured in a conventional drying machine (Benz, TKF 15/M350 +LFV/2 350R) at 150 °C for 2.5 min. Some of the samples were subjected to the textile care process by ISO 15797, in the Wascator FOM71 CLS, (Electrolux, Stockholm, Sweden), with the addition of 80 g of reference detergent 88060-A1 with optical brightener and 40 mL peroxyacetic acid. The washed samples were air-dried and subjected to tests. Sample labels and descriptions are presented in Table 2.

### 2.2. Methods

Field emission scanning electron microscopy (FE-SEM, Tescan, Czech Republic, FE-SEM, Mira II LMU) was used to characterise the surface morphology and to cover cellulose fibres with the chitosan. Prior to imaging on the FE-SEM, the samples were coated with chromium for 180 s in a steamer, Quorum Technologies, Q150T ES Sputter Coater, Laughton, UK.

Untreated and treated samples before and after the care process were analysed employing Fourier transform infrared spectroscopy (FTIR, PerkinElmer, Spectrum 100S, Shelton, CT, USA) using the attenuated total reflection (ATR) measurement technique, and the obtained spectral curves were processed in a Spectrum 100. Four scans were performed for each sample with the resolution of 4 cm^−1^ between 4000 cm^−1^ and 380 cm^−1^.

The contact angle was measured on a drop shape analyser, KRÜSS GmbH, DSA30S, Hamburg, Germany. The standard configuration of the DSA30 instrument is designed for semi-automatic contact angle measurements. The test was carried out according to the ASTM D7334. A fabric sample was placed on a table captured by a camera directly connected to a computer monitor, and a drop of distilled water at room temperature was placed on the fabric surface using a syringe. Distilled water that fell in the form of a drop on the textile had a volume of 10 µL (not determined by the program). The DSA30S software automatically started the measurement process directly after dosing the liquid, and the data were recorded as soon as the drop touched the surface of the textile. The ADVANCE program measured the left (l) and right (r) angles of the drop and calculated the mean value of both as well.

To monitor the durability of the treatment and determine the presence of chitosan particles bonded to the cotton materials in plain and satin weave, the mass per unit area was determined after the treatment and care cycle of the treated samples according to ISO 3801:1977.

Antimicrobial activity was determined according to the AATCC TM 147-2016, Antibacterial Activity Assessment of Textile Materials: Parallel Streak Method. Efficacy was determined on Gram-positive bacterium *Staphylococcus aureus* ATCC 6538 (*S. aureus*), Gram-negative *Escherichia coli* ATCC 8739 (*E. coli*), and *Candida albicans* ATCC 10231 (*C. albicans*).

## 3. Results and Discussion

### 3.1. Morphological Characterization of the Sample Surfaces

**Figure 1:**
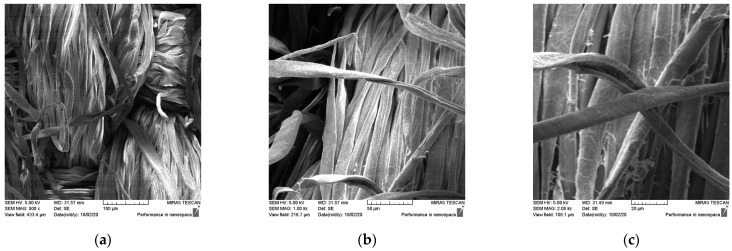
SEM micrographs of untreated plain weave cotton fabric at the magnifications of: (**a**) 500x, (**b**) 1000x, and (**c**) 2000x.

**Figure 2:**
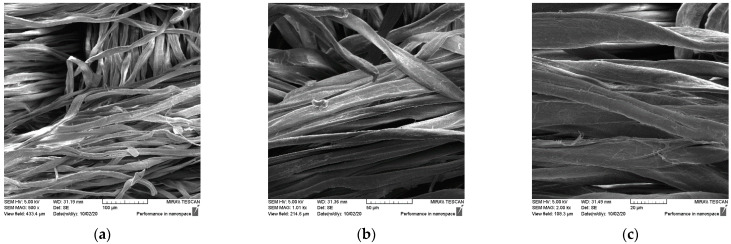
SEM micrographs of untreated satin weave cotton fabric at different magnifications: (**a**) 500x, (**b**) 1000x, and (**c**) 2000x.

**Figure 3:**
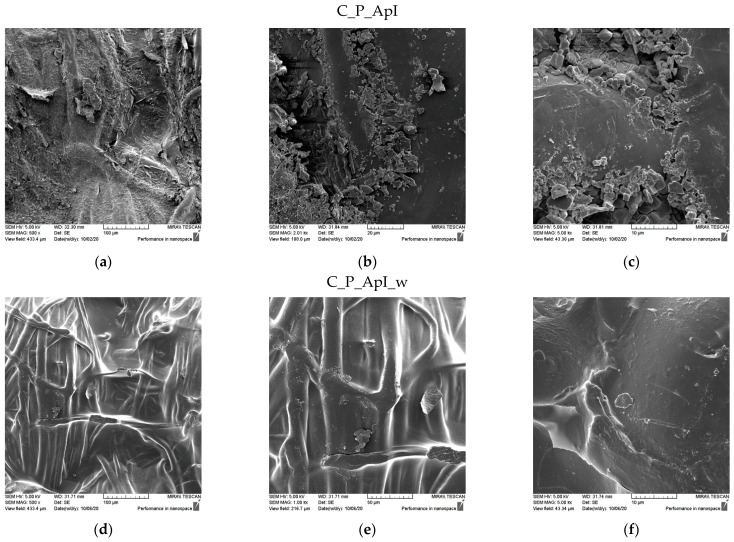
SEM micrographs of plain weave fabric samples treated with Bath I before (C_P_ApI) and after washing (C_P_ApI_w) at different magnifications: (**a**,**d**) 500x, (**b**,**e**) 2000x, and (**c**,**f**) 5000x.

**Figure 4:**
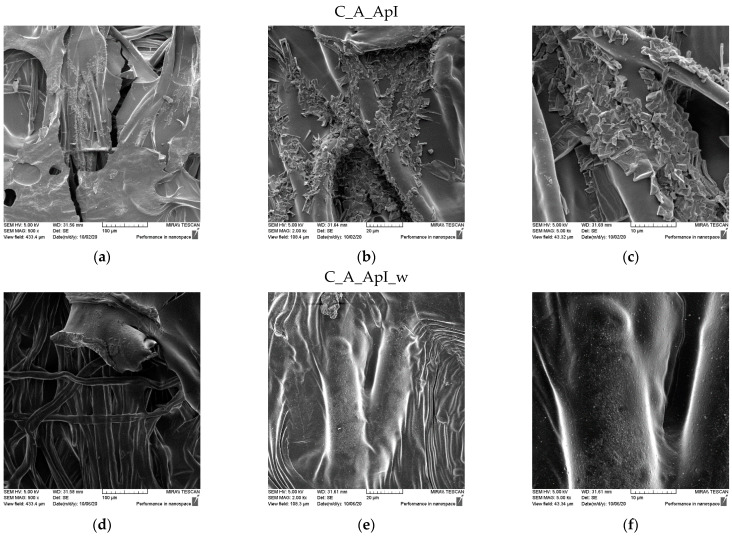
SEM micrographs of satin weave fabric samples treated with Bath I before (C_A_ApI) and after washing (C_A_ApI_w) at different magnifications: (**a**,**d**) 500x, (**b**,**e**) 2000x, and (**c**,**f**) 5000x.

**Figure 5:**
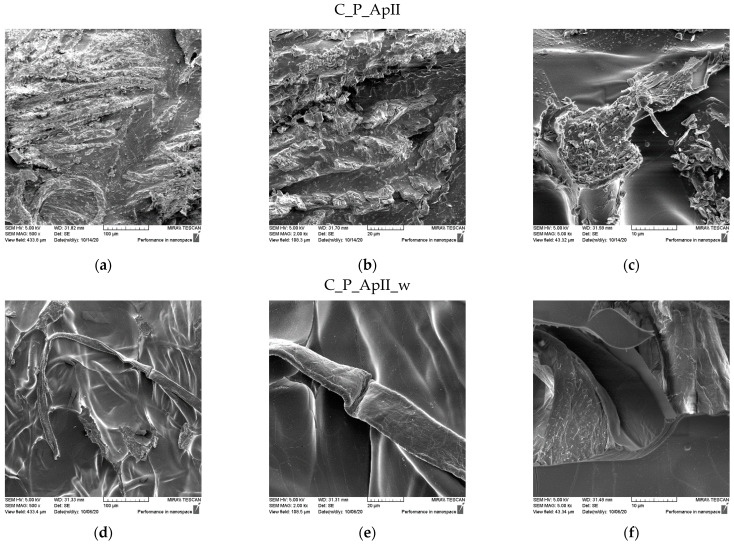
SEM micrographs of plain weave fabric samples treated with Bath II before (C_P_ApII) and after washing (C_P_ApII_w) at different magnifications: (**a**,**d**) 500x, (**b**,**e**) 2000x, and (**c**,**f**) 5000x.

**Figure 6:**
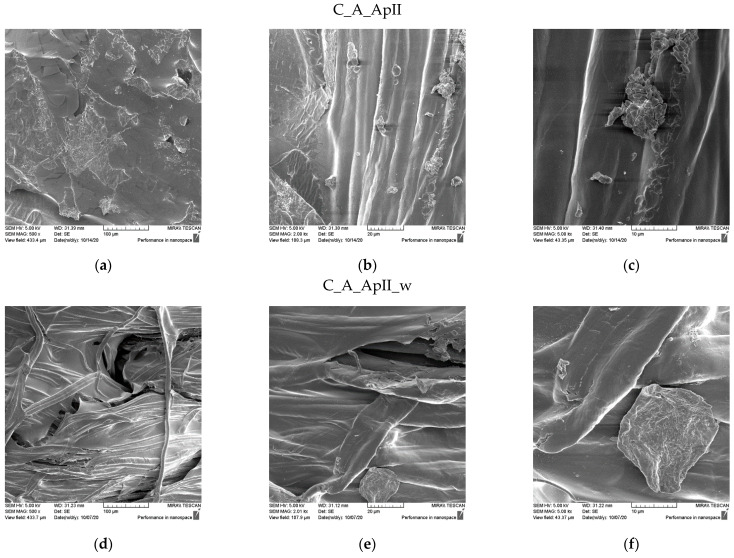
SEM micrographs of satin weave fabric samples treated with Bath II before (C_A_ApII) and after washing (C_A_ApII_w) at different magnifications: (**a**,**d**) 500x, (**b**,**e**) 2000x, and (**c**,**f**) 5000x.

Field emission scanning electron microscopy was used for morphological characterization of textile fabrics before (Figure 1 and Figure 2) and after treatment and care cycles (Figure 3, Figure 4, Figure 5 and Figure 6). In order to see the coverage and presence of chitosan particles on the samples, the samples were observed at three different magnifications. Figure 1 and Figure 2 show SEM micrographs of the surface morphology of untreated standard cotton fabric in plain (CO_P) and satin weave (CO_A) at different magnifications. The micrographs show a smooth surface of fibres with a characteristic twisted ribbon, less fibrillation is visible in the higher magnification microscopic images (Figure 1c and Figure 2c).

The SEM micrographs of the surfaces of the samples after treatment in Bath I and after the care cycles performed are shown for the plain weave sample in Figure 3 and for the satin weave sample in Figure 4. the presence of chitosan agglomerates with a slightly higher coating amount on a plain weave fabric sample can be seen (Figure 3) from the micrograph in Figure 3a–c and Figure 4a–c. The reason for the better retention of chitosan particles with tea tree essential oil on the surface of the plain weave sample was the density of the fabric, which had a greater number of contact or weaving points. Additionally, over the entire surface of both samples, the coverage of the fibre surface with the film that formed during the hydrothermal synthesis of the chitosan and tea tree essential oil on cellulose with maleic acid as a crosslinker was visible. On both samples in the plain (Figure 3d–f) and satin weave (Figure 4d–f) after the textile care cycle, a reduced presence of chitosan particles was observed, but the whole surface was still covered with a film and it was not possible to see the characteristic twisted ribbon of the structure of cellulose fabrics.

SEM micrographs of the samples of plain weave (Figure 5) and satin weave (Figure 6) cotton fabrics treated with Bath II before and after the care cycle are also shown. As with the samples treated in Bath I, the plain weave samples on the surface contained significantly higher amounts of agglomerates and chitosan particles, and in both samples, the fibre surface was completely covered with a chitosan film before and after the care cycle (Figure 5 and Figure 6). The satin weave samples treated with Bath II had a solid film on the surface, and at smaller amounts of chitosan, agglomerates were visible at 2000x and 5000x magnification. After washing, these agglomerates were still present, however, in smaller quantities. The film covering the fibres was still present, and the characteristic twisted ribbon of cotton fibres could be recognised in some places. The patterns in the fabric weave had much more pronounced chitosan agglomerates on the surface, which we associated with the density of the threads moving less during washing due to a larger number of weaving points. By washing, unbound chitosan particles were torn off and the coverage of the fibres with the chitosan film became visible.

### 3.2. Physico-Chemical Characterization of the Samples Established by Spectroscopy with Fourier Transform of the Infrared Spectrum

**Figure 7:**
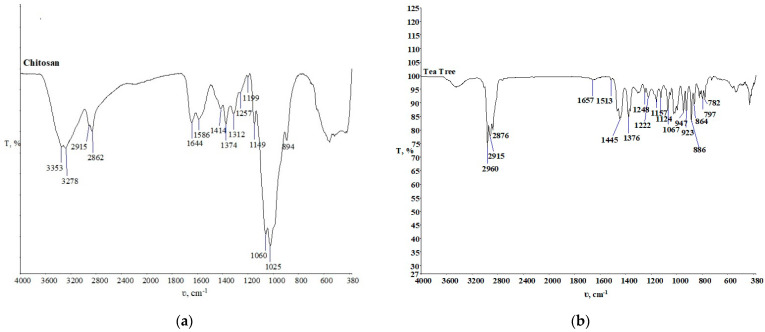
FTIR spectra of chitosan (**a**) and tea tree essential oil (**b**).

**Figure 8:**
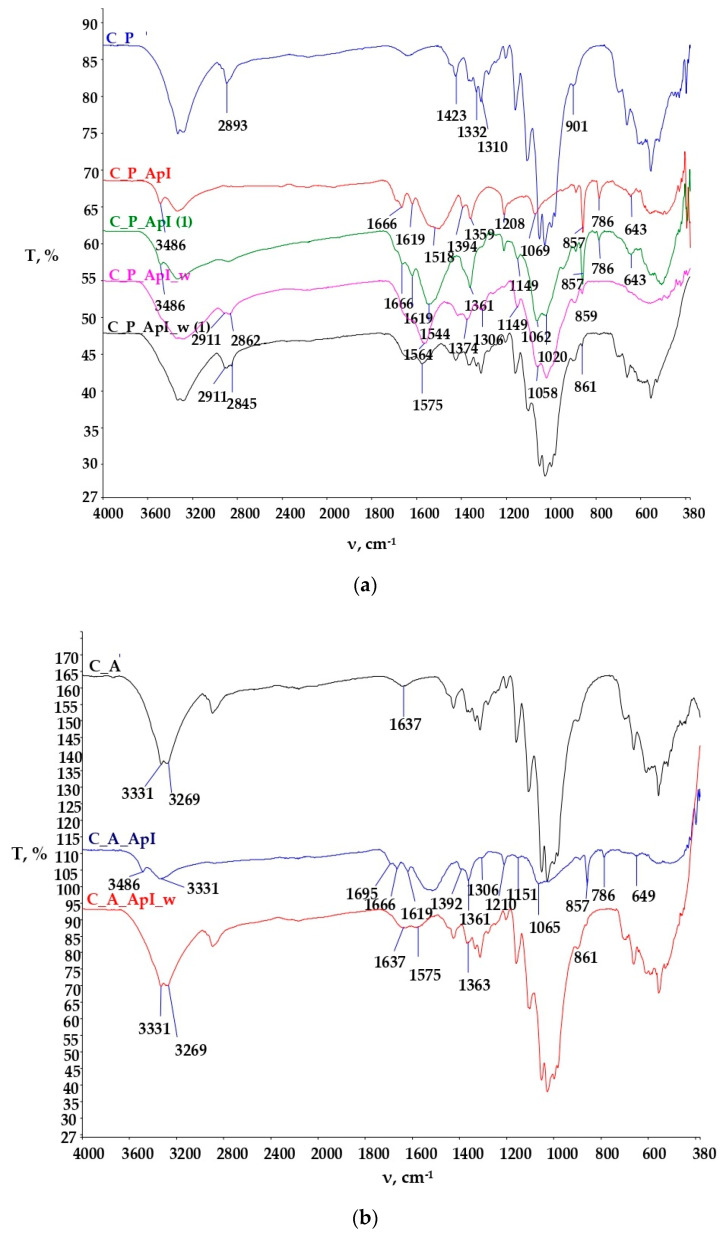
FTIR-ATR spectral curves of the untreated cotton sample and the sample treated in ApI before and after the care cycle (ApI_w): (**a**) in the plain weave (C_P), (**b**) in the satin weave (C_A).

**Figure 9:**
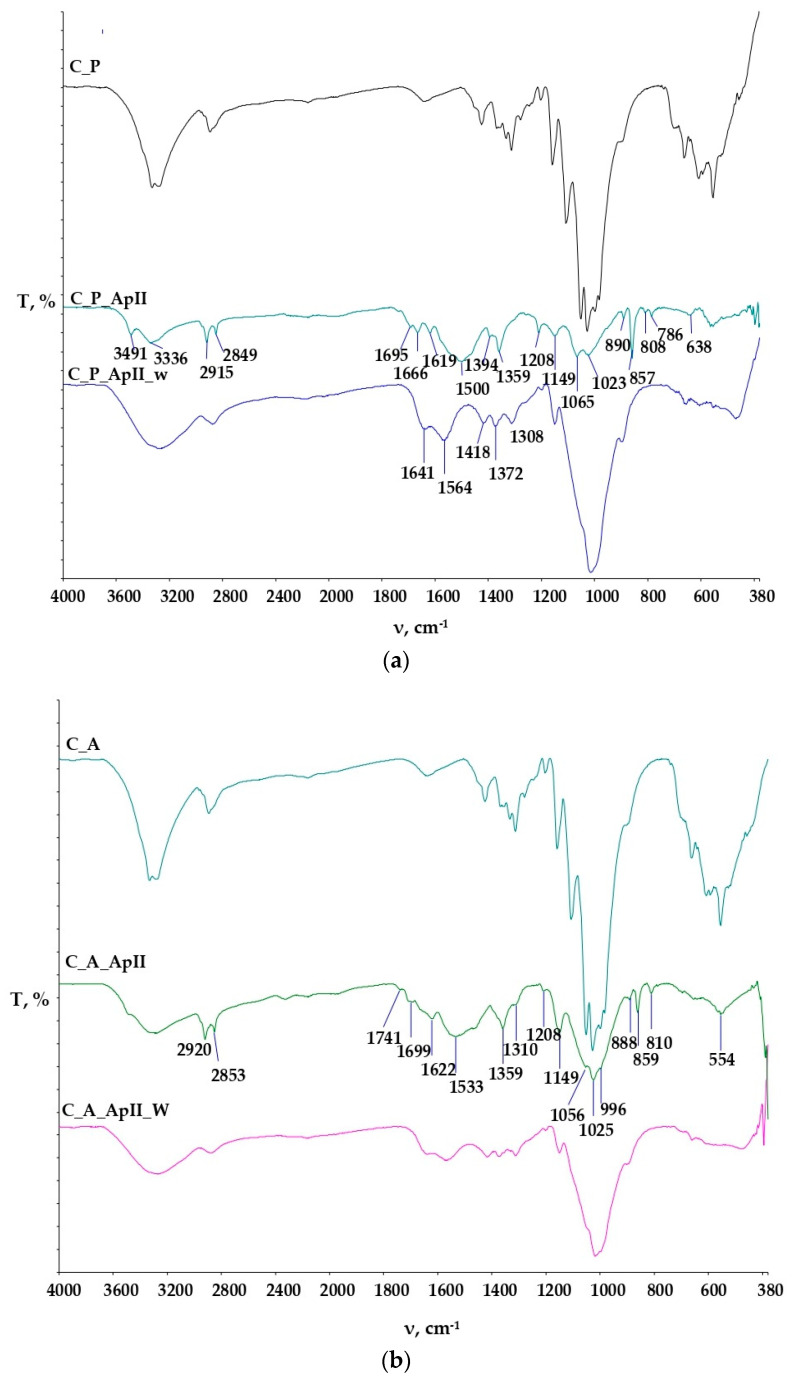
FTIR-ATR spectral curves of the untreated cotton sample and the sample treated in ApII before and after the care cycle (ApII_w): (**a**) In the plain weave (C_P), (**b**) in the satin weave (C_A).

**Figure 10:**
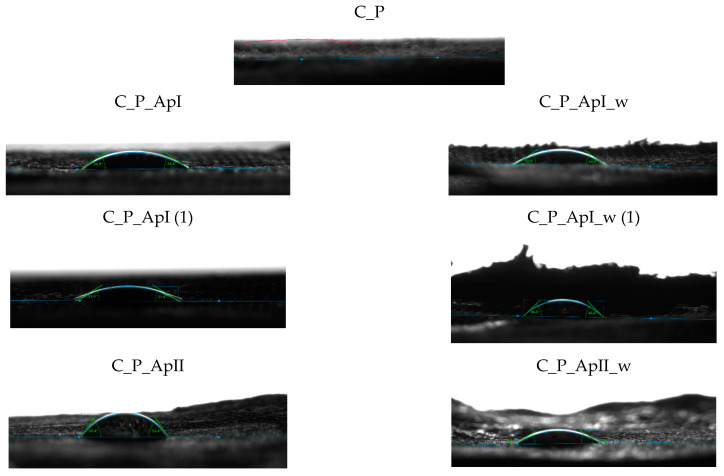
Photographs of a water drop on the plain weave fabric samples during contact angle measurement.

**Figure 11:**
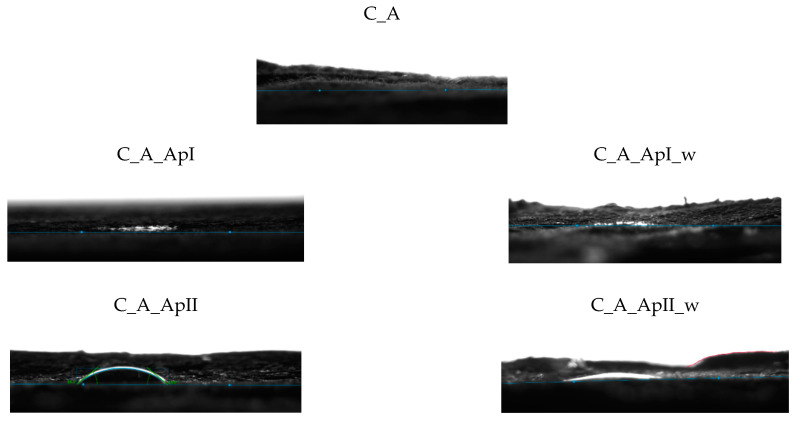
Photographs of a water drop on the satin weave fabric samples during contact angle measurement.

The physico-chemical characteristics of the pure components, the untreated cotton materials, and the treated cotton materials were investigated using Fourier transform infrared spectroscopy (FTIR) (Perkin Elmer, Shelton, CT, USA, Software Spectrum 100), and the spectra obtained are shown in Figure 7, Figure 8, Figure 9, Figure 10 and Figure 11.

Figure 7a shows the chitosan FTIR spectra for further monitoring and comparison with the FTIR spectra of the treated samples before and after the textile care procedure. Since it is a polysaccharide, considerable similarities with the cellulose FTIR spectra can be seen [19]. The FTIR spectrum of tea tree essential oil is shown in Figure 7b. It shows numerous peaks in the wave range from 1700 cm^−1^ to 600 cm^−1^. It is known that in the range of wavenumbers 889, 864, and 799 cm^−1^ the peaks indicate the presence of terpinene-4-ol, and the peaks present at wavenumbers 830, 815, and 780 cm^−1^ indicate the presence of γ-terpinene [23].

The spectral curves of the samples of plain and satin weave cotton fabrics untreated and treated in Bath I and II are shown in Figure 8 and Figure 9.

Figure 8a shows the spectra of the plain weave cotton fabric untreated and treated with Bath I before and after care cycles. The FTIR spectra of the C_P_ApI sample in the peak at 3486 cm^−1^ indicate N-H stretching due to the presence of chitosan. The presence of peaks is still visible after textile care cycles t, but at a significantly reduced intensity. Peaks in the range 1666 cm^−1^ to 1619 cm^−1^ indicate the vibrational stretching of C=O and NH groups in NHCOCH_3_ [23], but these peaks disappeared during the textile care cycle. Peaks at 1544 cm^−1^, 1564 cm^−1^, and 1575 cm^−1^ indicate the vibrational stretching of C=O groups, most likely due to the presence of carboxylate anions from maleic acid. Peaks 1069 cm^−1^, 1062 cm^−1^, and 1058 cm^−1^ indicate C-O vibrational stretching of chitosan [23,24,25,26,27,28,29]. Peaks at 857 cm^−1^, 786 cm^−1^, and 643 cm^−1^ indicate the absorption signals of monosubstituted benzenes, i.e., the presence of terpinen-4-ol and γ-terpinene from tea tree oil [27]. The peaks at the wavenumbers 859 cm^−1^ and 861 cm^−1^ on the FTIR spectra after the care cycle (C_P_ApI_w, C_P_ApI_w (1)) were significantly reduced in intensity, but still present, indicating the binding of chitosan prepared with tea tree oil in the structure of the cellulosic material. The samples were monitored at two locations and their adsorption curves are shown to differ from each other due to the surface irregularity of the treated samples before and after the care cycle.

Figure 8b shows the FTIR spectra of the untreated satin weave cotton fabric (C_A), and the FTIR spectra of the sample treated with Bath I before (C_A_ApI) and after care cycles (C_A_ApI_w). The FTIR spectrum of the sample C_A_ApI indicates the appearance of stretching and the symmetrical flexion of characteristic groups for polysaccharides at wavenumbers of 3331 cm^−1^, 1695 cm^−1^, 1666 cm^−1^, and 1619 cm^−1^. After the care cycle, peaks at wavenumbers of 1637 cm^−1^ and 1575 cm^−1^_,_ which occurred due to the stretching of carbonyl groups (ketones, aldehydes, and esters) are visible in the FTIR spectra of the C_A_ApI_w samples, exhibiting the crosslinking of chitosan with cellulose via maleic acid. In addition, physicochemical changes in the sample structure were clearly visible in the spectral curves between the untreated C_A and the treated sample C_A_ApI, at the peak 1210 cm^−1^, resulting from the elongation of the CO bond, which indicates the aromatic ester or alkyl aryl ether, the peak at 1151 cm^−1^ occurred due to the symmetrical elongation of COC bonds and the peak at 857 cm^−1^ arose as the result of absorption signals of monosubstituted benzenes and the presence of terpinen-4-ol and γ-terpinene from tea tree oil [25]. Peaks at wavenumbers 857 and 786 cm^−1^ were visible in the treated sample after care cycles, which confirmed the presence of terpinen-4-ol and γ-terpinen, important constituents of tea tree essential oil. After the care cycle (C_A_ApI_w), there was a decrease in the intensity of individual peaks, shifts, and disappearances of some peaks, indicating poorer stability of the binding of chitosan to cellulose on the sample in the satin weave [18,19,20,21,22,23,24,25,26,27].

Figure 9 shows the FTIR spectra of the cotton material in the plain (Figure 9a) and satin weave (Figure 9b) treated with Bath II before and after the care cycle, as well as the untreated cotton fabrics. The FTIR spectra of the treated samples (C_P_ApII, C_A_ApII) have a lot of new peaks as compared to the untreated sample. The peaks at the wavenumbers 3491 cm^−1^, 3336 cm^−1^ (C_P_ApII), and 3309 cm^−1^ (C_A_ApII) indicate the vibrational elongation of C=O and OH groups and NH groups characteristic of the polysaccharide structure, i.e., the structure of chitosan, but after the care cycle they were not visible. Additionally, peaks 2920 cm^−1^ and 2853 cm^−1^ indicate vibrational stretching within the C-H group. Peaks 1533 cm^−1^, 1622 cm^−1^, 1699 cm^−1^, and, 1741 cm^−1^ indicate the presence of carbonyl compounds, i.e., the presence of ester bonds, pointing at the binding of chitosan to cellulose by maleic acid. After care cycles, there was a shift in the peak from the wavenumber 2853 cm^−1^ to 2909 cm^−1^, which indicates the overlap of CH=O and CH vibrational stretching from cotton and chitosan. Peaks at 1412 cm^−1^ and 1372 cm^−1^ indicate C-H vibrational bending. The peak at 1730 cm^−1^ and 1562 cm^−1^ (Figure 9b. C_A_ApII_w) represents ester bonds and carbonyl groups from the acid, respectively. It is clear from all this that the successful binding of chitosan to the structure of the cellulose material took place [24,25,26,27,28,29,30,31,32,33,34,35,36]. Table 3 and Table 4 show the values of measurements made with a goniometer using droplet analysis and a tilting table (DSA30S). The CA (contact angle) marked the contact angle in the middle (m), on the left (l), and on the right (r). Each sample was measured twice, and both results were entered when there were differences between them. The measurements were made immediately after the contact of the droplet to the textile surface, while the contact angle was measured until the droplet was completely absorbed into the textile material.

The results of the contact angles for plain weave fabrics (Table 3) after treatment in Bath I and II are higher than those for satin weave fabrics (Table 4). The reason for this may be higher thread density, i.e., a greater number of contact points in the plain weave samples, due to which the capillary forces were lower, but also the presence of more chitosan particles on the chitosan cover layer on the samples after care cycles (Figure 3 and Figure 5), as opposed to the samples in satin weave after care cycles (Figure 4 and Figure 6). The photographs of the untreated and the samples treated in Bath I and Bath II before and after care cycles immediately after the contact of the drop with the textile material when testing the contact angle on the plain weave and satin weave samples are shown in Figure 10 and Figure 11.

The satin weave fabric samples treated before and after the care cycle usually absorbed the drop of distilled water immediately. The exception was the sample treated in Bath II, where a minimum time was required for the droplet to be absorbed, and this can also be seen in Figure 11 for the samples C_A_ApII, where the visible liquid was spilled on the surface of the material. The reason for the short retention of the water droplet on the sample surface was the chitosan film with which the fibres were coated and which is also visible in the SEM micrographs. From the measurements carried out on the samples C_P_ApI_w and C_P_ApI_w (1), it could be seen that the sample behaved differently at different measuring points, which indicates uneven surface treatment. The results of the measured contact angles of the samples treated in Bath I were obtained only for the sample in the plain weave, while the highest contact angle was measured on the sample C_P_ApI_w (1), amounting to 36.825°. A higher contact angle was measured in both the tested plain weave fabric samples treated in Bath I after the care cycle C_P_ApI_w and C_P_ApI_w (1) compared to the unwashed samples (C_P_ApI, C_P_ApI (1)) presented in Table 5. The reason for this could be the removal of chitosan agglomerates from the surface, which due to their ionic structure could be protonated with H+ in water to form a cationic polyelectrolyte, thus increasing the absorbency of the water [34,35].

In addition, the fabric shrank in the care cycle and the SEM micrographs (Figure 3) showing that the fibres were covered with a chitosan film. The satin weave samples treated in Bath I absorbed water and the contact angle values could not be measured. From the results obtained for the sample treated in Bath II (Table 2 and Table 4) before and after the care process, the largest contact angle was recorded for the plain weave sample C_P_ApII (30.52°), followed by C_A_ApII (27.74°) and C_P_ApII_w (22.25°).

In the case of sample C_A_ApII_w, the contact angle was not measured due to the rate of droplet penetration into the sample. Table 3 and Table 5 show standard deviation of the results of the contact angles measured for the samples treated with Baths I and II, and it can be seen that the smallest deviations occurred in the sample C_P_ApI_w, and the largest in the sample C_P_ApII. Since the contact angle (left, right, and middle) was the same for all the three samples, there were no deviations in the values within each individual sample. Regardless of some differences in the contact angles shown in the figures, it can be concluded that all the samples were hydrophilic, as the longest absorption time of the droplet in the textile material was only 18 s, while most samples did not even form a dome on the material. The hydrophilicity of the material could also be seen in the shape of the dome and the value of the contact angle, which was quite low.

The change in the mass per unit area of the samples was calculated according to ISO 3801-1977 as per method 5. The weight difference and percent of weight change of the samples after finishing were calculated in relation to the untreated fabric. Additionally, weight difference and percent of weight change of the treated samples after the care cycle were calculated in relation to the untreated samples after the care cycle in order to cancel out the influence of shrinkage on mass gain. From the values obtained in Table 5, it can be seen that all the samples showed an increase in mass per unit area after the treatments, with the greatest increase in the sample C_A_ApI (34.16%). After the care cycle C_A_ApI_w, a large decrease in mass per unit area (10.00%) was observed compared to the same sample before the care cycle, indicating the removal of unbound chitosan particles from the sample surface. From the results obtained, it can be seen that the percentage of mass increase in plain weave samples treated before (C_P_ApII, 28.62%) and after the care cycle (C_P_ApII_w, 25.15%) in Bath II changed slightly and was extremely high compared to the untreated samples. Considering the mass change after the care cycle of the untreated sample (C_P_w, 0.47%), which was due to the shrinkage of the fabric, it can be concluded that the binding of the chitosan particles to the structure of the cellulosic material in the plain weave was successfully accomplished. Values of standard deviation obtained showed larger differences in the untreated and treated samples before and after the care cycle in satin weave compared to the samples in plain weave, which was expected precisely because of the previously mentioned features of yarn interlacing.

From the antimicrobial activity results presented in Table 6, it can be seen that the treated samples showed a bacteriostatic effect, as no microorganisms developed on or under them.

**Figure 12:**
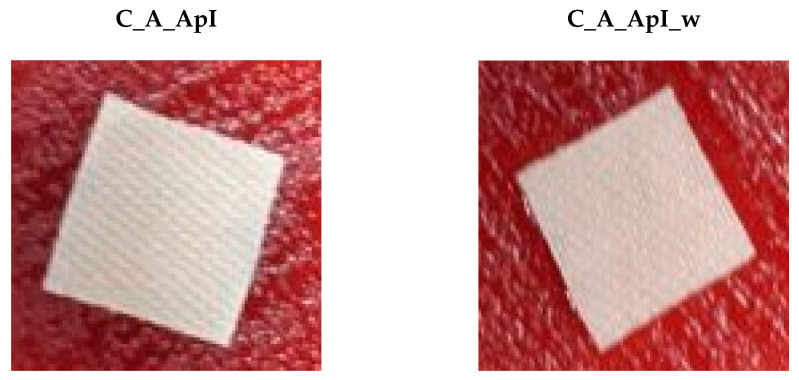
Photographs of antimicrobial testing on *Candida albicans* samples of cotton fabric in satin weave treated in Bath I before (C_A_ApI) and after the care cycle (C_A_ApI_w).

The untreated samples (C_P, C_A) exhibited no antimicrobial activity. The samples in satin treated in Bath I showed effective antifungal activity with a 1 mm zone of inhibition on *Candida albicans* (Figure 12). The antimicrobial efficacy of both treated samples in Baths I and II could be related to the polycationic nature of chitosan, which interfered with bacterial metabolism and thus prevented their growth and development on the treated tissue [36]. In addition to the above action of chitosan, enhanced effects of antifungal efficacy were seen with the addition of tea tree essential oil, which is known to have antifungal activity by altering membrane properties and compromising membrane-related functions [37]. Considering the overall results, we can conclude that the samples treated with Bath I showed better antimicrobial properties. Additionally, we can conclude that almost all the samples exhibited bacteriostatic efficiency, as although the zone of inhibition could not be observed, no colonies beneath or on the surface of the samples were recorded.

Considering the final purpose of the prepared samples for medical compresses, mechanical properties and aesthetic components related to the change in whiteness or yellowing of the samples do not represent a significant requirement and were not evaluated in this research.

## 4. Conclusions

Chitosan is not only used to achieve antimicrobial resistance in textiles, but also in industrial processes as a coagulant, as well as for broader medical applications. Chitosan found its application in numerous branches of human activity, and due to its properties and antimicrobial effectiveness, it is an interesting substance to be used for medical and wellness purposes. The possibility of using tea tree essential oil with chitosan with the aim of improving the antimicrobial effect of cotton fabrics in two different weaves, linen and satin, was investigated. Emphasis was placed on the possibility of achieving stable bonds between cellulose material and chitosan with and without the addition of tea tree essential oil, using maleic acid as a crosslinker and solvent, and applying in situ hydrothermal synthesis. The obtained micrographs of the morphology surface of the samples on FE-SEM showed the coverage of the sample with a thin chitosan film in the form of a larger amount of agglomerates in the plain weave fabric samples. After the care cycle, the agglomerates were removed from the surfaces of the samples, but a film covering the fibres was still visible in all the samples, which indicated stability of the treatment for the samples in Baths I and II, to which the process of in situ hydrothermal synthesis definitely contributed. The FTIR spectra obtained by FTIR-ATR analysis indicate physicochemical changes in the structure of all the samples in plain and satin weave after the treatments in Baths I and II and after the care cycle, proving the treatments were durable.

Analysis of the samples on the goniometer proved the hydrophilicity of the treated fabrics, despite the film that had formed on the surface of the samples. The satin weave fabric samples treated in Baths I and II showed higher hydrophilicity compared to the plain weave fabric samples, which is to be expected given the structural differences between the two weaves. In the plain weave, each weft thread was interlaced with each warp thread. This created a large number of “channels” in which chitosan particles could remain. In the satin weave, the warp thread interlaces with every fifth weft thread. There is a larger flat area between the threads and the finish is easier to remove from the surface since there are no indentations, as there are in plain weave fabrics. Hydrophilicity is a desirable property for materials used as a medical compress.

Previously mentioned structural differences of plain weave and satin weave fabrics are the reason for the measured differences in the mass per unit area of the treated samples after the care cycle compared to the same untreated sample before and after the care cycle. From the mass per unit area results, it can be seen that the increase in the mass of the treated samples after the care cycle was more stable for the plain weave samples than for the satin weave samples. Antimicrobial efficacy against bacteria and fungi was confirmed in all the samples treated in Baths I and II before and after the care cycle. It should be emphasised that the best antimicrobial effect was recorded in the samples of the satin bond treated in Bath I before and after the care cycle (C_A_ApI and C_A_ApI_w), where resistance to the *Candida albicans* fungus was proven with an inhibition zone of 1 mm visible. The results obtained during the research provide sets guidelines for the further development of environmentally acceptable in situ processing of cellulose material with chitosan while optimizing the amount of essential tea tree oil, as well as the application of other active ingredients with known antimicrobial or wellness effects.

## Figures and Tables

**Table 1:** Bath composition.

Ingredients	Bath I (ApI)	Bath II (ApII)
Maleic acid (Sigma Aldrich, Spain)	10 g/L	
Sodium hypophosphite monohydrate (Sigma Aldrich, Stockholm, Sweden)	6 g/L	
Chitosan (Tricomed SA, Łódź, Poland)	50% by weight of material	
Tea Tree Oil (Sigma Aldrich, Stockholm, Sweden)	40% tea tree oil by weight of chitosan	-

Deionised water was used to prepare both baths.

**Table 2:** Sample label.

C_P	cotton sample in plain weave
C_P_w	cotton sample in plain weave, and washed once
C_A	cotton sample in satin weave
C_A_w	cotton sample in satin weave and washed once
C_P_API	cotton sample in plain weave, treated with Bath I in an autoclave at 80 °C, 24 h
C_P_API_w	cotton sample in plain weave, treated with Bath I in an autoclave at 80 °C, 24 h, and washed once
C_A _API	cotton sample in satin weave, treated with Bath I in an autoclave at 80 °C, 24 h
C_A_API_w	cotton sample in satin weave, treated with Bath I in an autoclave at 80 °C, 24 h, and washed once
C_P_APII	cotton sample in plain weave, treated with Bath II in an autoclave at 80 °C, 24 h
C_P_APII_w	cotton sample in plain weave, treated with Bath II in an autoclave at 80 °C, 24 h, and washed once
C_A_APII	cotton sample in satin weave, treated with Bath II in an autoclave at 80 °C, 24 h
C_A_APII_w	cotton sample in satin weave, treated with Bath II in an autoclave at 80 °C, 24 h, and washed once

**Table 3:** Results of the contact angle with standard deviations of the contact angle for the samples in plain weave.

Samples	CA (m)	CA (l)	CA (r)	Temp	Mean Three-Phase Point (l)/(mm)	Mean Three-Phase Point (r)/(mm)	Mean Diameter (mm)	Mean Volume (µL)
C_P	0	0	0	0	0	0	0	0
C_P_ApI	19.0 ± 4.5	19.0 ± 4.5	19.0 ± 4.5	20	12 ± 0.2	30.8 ± 0.7	18.8 ± 0.7	228.3 ± 78.5
C_P_ApI (1)	24.0 ± 6.1	24.0 ± 6.1	24.0 ± 6.1	20	13.1 ± 1.1	33 ± 0.2	19.8 ± 1.1	324.4 ± 64.1
C_P_ApI_w	27.3 ± 4.4	27.3 ± 4.4	27.3 ± 4.4	20	14.1 ± 0.9	30.4 ± 0.3	16.3 ± 1.2	219.6 ± 83.3
C_P_ApI_w (1)	36.8 ± 5.3	36.8 ± 5.3	36.8 ± 5.3	20	14.2 ± 0.2	29.8 ± 0.1	15.6 ± 0.2	260.9 ± 49.2
C_P_ApII	30.5 ± 14.0	30.5 ± 14.0	30.5 ± 14.0	20	11 ± 2.2	30.4 ± 0.7	19.5 ± 2.9	360.1 ± 57.4
C_P_ApII_w	22.3 ± 6.0	22.3 ± 6.0	22.3 ± 6.0	20	12.8 ± 0.2	29.2 ± 0.1	16.5 ± 0.3	177.4 ± 56.3

(1) designation of the second measuring position of the sample C_P_API.

**Table 4:** Results of the contact angle (CA) and standard deviations of the contact angle for the samples in satin weave.

Samples	CA (m)	CA (l)	CA (r)	Temp	Mean Three-Phase Point (l)/(mm)	Mean Three-Phase Point (r)/(mm)	Mean Diameter (mm)	Mean Volume (µL)
C_A	0	0	0	0	0	0	0	0
C_A_ApI	0	0	0	0	0	0	0	0
C_A_ApI_w	0	0	0	0	0	0	0	0
C_A_ApII	27.7 ± 7.7	27.7 ± 7.7	27.7 ± 7.7	20	11.8 ± 0.1	27.8 ± 0.2	16.0 ± 0.2	205.4 ± 69.9
C_A_ApII_w	0	0	0	0	0	0	0	0

**Table 5:** The change in the mass per unit area of the samples.

Samples	Mass per Unit Area, g/m^2^ *	STDEV (σ), g/m^2^	Weight Difference, g	Weight Change, %
C_P	158.3 ± 0.4	0.4		
C_P_w	159.0 ± 0.7	0.7	0.74	0.47
C_P_API	206.0 ± 2.0	2.0	47.65	30.11
C_P_API_w	185.9 ± 0.6	0.6	26.91	16.93
C_P_APII	204.5 ± 0.6	0.6	45.51	28.62
C_P_APII_w	198.1 ± 0.7	0.7	39.8	25.15
C_A	178.5 ± 1.9	1.9		
C_A_w	196.5 ± 2.2	2.2	18	10.09
C_A_API	239.4 ± 1.8	1.8	60.95	34.16
C_A_API_w	216.1 ± 1.7	1.7	19.65	10.00
C_A_APII	211.1 ± 2.6	2.6	32.65	18.30
C_A_APII_w	203.9 ± 0.2	0.2	7.45	3.79

* Average value of 5 measurements.

**Table 6:** Results of antimicrobial activity for an untreated and treated sample before and after care cycles.

Samples	*Staphylococcus aureus*	*Escherichia coli*	*Candida albicans*
C_P	−	−	−
C_A	−	−	−
C_P_ApI	+/−	+/−	+/−
C_P_ApI_w	+/−	+/−	+/−
C_A_ApI	+/−	+/−	+
C_A_ApI_w	+/−	+/−	+
C_P_ApII	+/−	+/−	+/−
C_P_ApII_w	+/−	+/−	+/−
C_A_ApII	+/−	+/−	+/−
C_A_ApII_w	+/−	+/−	+/−

+ antimicrobial activity (zone of inhibition can be observed), +/− partial antimicrobial activity (zone of inhibition cannot be observed, but no colonies beneath), − no antimicrobial activity.

## Data Availability

The data presented in this study are available on request from the corresponding author.
